# Epidemiology, management, complications and costs associated with type 2 diabetes in Brazil: a comprehensive literature review

**DOI:** 10.1186/1744-8603-9-62

**Published:** 2013-12-03

**Authors:** Andréa D Bertoldi, Panos Kanavos, Giovanny V A França, André Carraro, Cesar Augusto Ovieda Tejada, Pedro C Hallal, Alessandra Ferrario, Maria Inês Schmidt

**Affiliations:** 1 Programa de Pós-graduação em Epidemiologia, Universidade Federal de Pelotas, Rua Marechal Deodoro, 1160 3° piso, Pelotas, RS, Brazil, 96.020-220; 2 LSE Health, London School of Economics and Political Science, London, UK; 3 Department of Social Policy, London School of Economics and Political Science, London, UK; 4 MRC Epidemiology Unit, Institute of Metabolic Science, Cambridge, UK; 5 Programa de Pós-Graduação em Organizações e Mercados, Universidade Federal de Pelotas, RS, Brazil; 6 Programa de Pós-graduação em Epidemiologia, Universidade Federal do Rio Grande do Sul, Porto Alegre, RS, Brazil

**Keywords:** Diabetes, Brazil, Costs, Epidemiology

## Abstract

**Background:**

With an estimated 74% of all deaths attributable to non-communicable diseases (NCDs) in 2010, NCDs have become a major health priority in Brazil. The objective of the study was to conduct a comprehensive literature review on diabetes in Brazil; specifically: the epidemiology of type 2 diabetes, the availability of national and regional sources of data (particularly in terms of direct and indirect costs) and health policies for the management of diabetes and its complications.

**Methods:**

A literature search was conducted using PubMed to identify articles containing information on diabetes in Brazil. Official documents from the Brazilian government and the World Health Organization, as well as other grey literature and official government websites were also reviewed.

**Results:**

From 2006 to 2010, an approximate 20% increase in the prevalence of self-reported diabetes was observed. In 2010, it was estimated that 6.3% of Brazilians aged 18 years or over had diabetes. Diabetes was estimated to be responsible for 278,778 years of potential life lost for every 100,000 people. In 2013, it is estimated that about 7% of patients with diabetes has had one or more of the following complications: diabetic foot ulcers, amputation, kidney disease, and fundus changes. The estimated annual direct cost of diabetes was USD $3.952 billion in 2000; the estimated annual indirect cost was USD $18.6 billion. The two main sources of data on diabetes are the information systems of the Ministry of Health and surveys. In the last few years, the Brazilian Ministry of Health has invested considerably in improving surveillance systems for NCDs as well as implementing specific programmes to improve diagnosis and access to treatment.

**Conclusions:**

Brazil has the capacity to address and respond to NCDs due to the leadership of the Ministry of Health in NCD prevention activities, including an integrated programme currently in place for diabetes. Strengthening the surveillance of NCDs is a national priority along with recognising the urgent need to invest in improving the coverage and quality of mortality data. It is also essential to conduct regular surveys of risk factors on a national scale in order to design effective preventive strategies.

## Background

Brazil is an upper middle-income country with a population of 190,755,799 inhabitants [1] and a per capita gross domestic product of USD $ 10,993 (current exchange rate) in 2011. With a land area covering 47% of Latin America [2], Brazil has marked regional inequalities in terms of climate, social development, income and other indicators.

Following democratisation of the country from 1994 onwards, Brazil has experienced a period of economic growth, which allowed the implementation of social development policies [3]. This has led to slow but stable improvements in social indicators, particularly reductions in poverty and in regional inequalities. In the 70s and early 80s, Brazil underwent a period of social mobilisation in which people campaigned for basic rights, including universal health care access. The demand for greater decentralisation of public resources led to an increase in the budget of cities and states. These factors contributed to the implementation of the Brazilian Unified Health System (SUS - *Sistema Único de Saúde*) in 1990 [4].

SUS is intended to provide healthcare free of charge to the whole Brazilian population, financed through direct and indirect sources such as tax revenues, social contributions, out-of-pocket spending, and employers’ health-care spending [5]. It includes primary health care units, hospitals, emergency departments, laboratories and blood centres. In 2006, SUS budget reached around USD $15 billion, which represents 54% of the country’s total health expenses [6]. Although access has expanded over the years, the increasing demands on SUS have had negative repercussions on the quality of the services delivered and on waiting times in hospitals and emergency departments [5].

In 2011, 22% of total health expenditure was spent on the payment of private health insurance [7]. The proportion of out-of-pocket expenses has continued to rise in spite of the implementation of SUS, from 9% in 1981 to 15% in 2003 and 19% in 2008 [5]. Out-of-pocket expenses are particularly concerning due to the difficulty in accurately predicting these costs [8] which can lead to catastrophic health spending. This is a problem affecting up to 16% of all Brazilian families [8-11].

Brazil and various other Latin American countries have undergone rapid demographic, epidemiological and nutritional transitions [12]. Dietary shifts towards low consumption of fiber and heavy consumption of saturated fatty acids and sugar and sedentary lifestyles are key contributors to the incidence of obesity, type 2 diabetes, and other chronic diseases [13]. Non-communicable diseases (NCDs) have become a major health priority in Brazil with an estimated 74% of all deaths attributable to NCDs in 2010 [14]. National estimates indicate that people with diabetes experience a 57% greater risk of death than the general population [15]. Beyond the health burden, diabetes is also responsible for increased use of health services and increased costs. Between 1999–2001, it was estimated that about 7.4% of all non-pregnancy related admissions to hospitals and 9.3% of all hospital costs in Brazil were attributable to diabetes [16].

In the present study we aimed to: (i) identify existing data sources on the prevalence of diabetes and its complications, as well as the direct and indirect costs of diabetes in Brazil; (ii) describe the prevalence of diabetes and its complications - retinopathy, nephropathy, neuropathy, diabetic foot ulcers, amputation, kidney disease, fundus changes, vascular complications; (iii) report evidence on direct and indirect costs; and (iv) review health policies for the management of diabetes and its complications.

### Methodology

A comprehensive literature search was conducted to identify articles containing information on type 2 diabetes in Brazil. The following PubMed search strategy was used: ("diabetes mellitus" [MeSH Terms] OR ("diabetes" [All Fields] AND "mellitus" [All Fields]) OR "diabetes mellitus" [All Fields] OR "diabetes" [All Fields] OR "diabetes insipidus" [MeSH Terms] OR ("diabetes" [All Fields] AND "insipidus" [All Fields]) OR "diabetes insipidus" [All Fields]) AND ("brazil" [MeSH Terms] OR "brazil" [All Fields]). The search was limited to articles published in Portuguese, English or Spanish between 2000 and October 2011, without any restrictions on the study design or the level (national or regional) at which the data were collected.

We included all publications providing information on one or more of the following end-points related to diabetes type 2 in Brazil: prevalence and incidence, management (treatment, access, and inequalities), complications (retinopathy, nephropathy, neuropathy, diabetic foot ulcers, amputation, kidney disease, fundus changes, vascular complications) and direct and indirect costs.

Articles were first screened by title and then by abstract. Full-text of selected publications were retrieved and examined regarding eligibility. Reference lists of the selected articles were scrutinized in order to identify relevant references. Official documents from the Brazilian government and the World Health Organization (WHO) were also examined. In addition, we identified unpublished work in the grey literature through Google, the researchers’ own knowledge and consultations with diabetes experts in Brazil.

## Results and discussion

We identified 2,699 articles published between 2000 and October 2011. The screening phase enabled us to identify 87 publications, which were retrieved for detailed evaluation. Forty-two publications met the eligibility criteria (Table 1).

**Table 1:** Literature review

**Area of diabetes management**	**Number of publications retrieved**	**References**
Prevalence, incidence and mortality	15	[17-30]
Prevalence and costs of complications	13	[15,17,21,31-40]
Management: treatment, access and inequality	6	[41-46]
Outcomes	6	[47-52]
Direct and indirect costs	7	[23,53-58]

### Data sources on diabetes in Brazil

The Ministry of Health has developed a comprehensive surveillance system for NCDs and their risk factors [17]. For diabetes, data is available on morbidity (Hospital Information Systems, Ambulatory Information System, and Hypertension and Diabetes Registration and Follow-up system), mortality (single cause or multiple causes) and risk factors (routine data collection through surveillance systems and surveys) [17].

The Hospital Information System (SIH-SUS) [17] is a national system that aggregates patient level data on hospital admissions, primary cause of admission, diagnosis, procedures, length of stay and reimbursement by SUS. The system is set up to allow download and tabulation of data at the municipal level. The scope of the system is limited to SUS admissions and does not include any information on admissions covered by private health insurance or paid out-of-pocket. It is estimated that SIH-SUS covers 60% to 70% of all hospital admissions in the country, although with large variations across regions.

As part of the Ambulatory Information System (SIA-SUS) [17] information is collected on so-called ‘highly complex procedures’. This includes data on treatment and exams in the areas of nephrology, cardiology, oncology, orthopaedics, ophthalmology among others. From this dataset it is possible to extract relevant information on screening and management of diabetes and its complications. For example, Georg et al. [59] performed an economic analysis using secondary data from the SIA-SUS (fasting plasma glucose measurement in order to confirm diagnosis of diabetes), aiming to estimate the cost-effectiveness of the screening programme for diabetes mellitus in Brazil.

The registration and follow-up system for hypertension and diabetes (HiperDia) [15] is a computerised system restricted to health system units that register prospective information on patients with hypertension and diabetes who are registered with a health unit or primary health care team. Aggregate data and reports are accessible online. This database includes information on the number of patients with hypertension, types 1 and 2 diabetes, the number of patients who are obese, smokers, physically inactive, as well as those diagnosed with other chronic complications (e.g. dyslipidaemia) [17]. It is estimated that 31.1% of patients with known diabetes in Brazil are registered in the HiperDia System [15]. However, there are concerns about the quality of the data. A recent study identified inaccuracies and contradictions in the information reported, indicating the need for additional training and more specific clinical and laboratory criteria to enhance identification of diabetes and hypertension-related complications [60].

The mortality information system (SIM) collects information on deaths nationwide [61]. The system includes reliable information on age, gender, place of residence and cause of death classified according to the International Classification of Diseases version 10 (ICD-10). Problems of misclassification regarding cause of death and coverage gaps are known in the north and northeast of the country [62]. However, even in these regions, major improvements have been documented in the recent years [17].

VIGITEL is a surveillance system of risk and protective factors for chronic NCDs through telephone interviews [63]. It was launched in 2006 in all capitals of the 26 Brazilian states including the Federal District and has been conducted since then on an annual basis. Each annual survey includes around 2,000 participants from each of the 27 capital cities with results weighted for the availability of land lines in each region.

The National Household Sample Survey (PNAD) provides periodic surveillance data on NCDs nationwide. Reports summarising data by region, by state, and by rural/urban area are accessible online [64]. The three surveys conducted to date provided information on access to and utilisation of health services in 1998, 2003 and 2008. In addition, the 2008 survey also included information on morbidities caused by chronic diseases, including diabetes [65].

The family budget survey (POF) [66] is a household survey measuring consumption, expenses and income of Brazilian families. Previous survey rounds were conducted in 1974/1975, 1987/1988, 1995/1996, 2002/2003 and 2008/2009. The survey provides information on the cost of treating diabetes, which allows for assessment of the disease’s impact on households budgets, for example [46].

The national demographic and health survey (PNDS) is part of the MEASURE DHS project [67], focusing on women of child-bearing age and children under five in Brazil. The PNDS was first conducted in 1986 and subsequently in 1996 and 2006; however, data on the prevalence of diabetes among women and access to medicines were only collected in 2006 [68].

The Brazilian longitudinal study of adult health (ELSA-Brasil) [69,70] is a multicentre cohort study funded by the Ministry of Health to investigate diabetes and cardiovascular disease (CVD) incidence, risk factors and complications. The baseline evaluation was completed in December 2010 and included 15,105 civil servants aged 35–74 years from six public universities in the northeast, south and southeast regions of Brazil. Annual telephone interviews are conducted to monitor the health status of each participant enrolled in the baseline [69].

### Prevalence of diabetes and diabetes-related mortality

In 2012, the International Diabetes Federation (IDF) estimated the prevalence of diabetes in Brazil to be 10.3% [71]. In the next paragraphs we summarise evidence on the prevalence of diabetes and diabetes-related mortality since 1986 across different regions in Brazil.

From 1986 to 1988, a multicentre study on diabetes was conducted in nine Brazilian state capitals, including a sample of 21,847 subjects first screened by fasting capillary glucose (FCG) [21]. The prevalence of diabetes was estimated at 7.6% among subjects aged 30–69 years. A concerning finding was that 46.5% of the cases were undiagnosed. In addition, out of those who were aware of their diabetes condition, 22.3% were not receiving any type of diabetes treatment. The prevalence of diabetes did not vary according to sex, ethnicity and level of education, but increased markedly with age, from 2.7% among those aged 30–39 years to 17.4% among those aged 60–69 years [21].

Since then, several other studies have been conducted with different scopes and methodologies, as summarised in Table 2. Most of the studies presented are based on self-reported diabetes.

**Table 2:** **Studies of the prevalence of diabetes in Brazil**^**1**^

**First author (year)**	**Site**	**Year of study**	**Sample size**	**Age group**	**Diabetes prevalence**	**Criteria**
Goldenberg (1996) [25]	São Paulo, SP	1986-1988	2,007	30-69 years	4.7%	Self-report
Malarbi (1992) [49]	Nine Brazilian	1988	21,847	30-69 years	7.6%	OGTT^2^ and self-report
	state capitals					
Torquato (2003) [30]	Ribeirão Preto, SP	1996-1997	1,473	30-69 years	12.1%	OGTT and self-report
Passos (2005) [27]	Bambuí, MG	1997	816 adults and 1,494 elderly	Adults (18-59 years); elderly (60+ years).	Elderly 14.6%	FPG^3^ and self-report
					Adults 2.3%	
Dias da Costa (2006) [24]	Pelotas, RS	2000	1,968	20-69 years	5.6%.	Self-report
Souza (2003) [29]	Campos dos Goytacazes, RJ	2001	1,039	>18 years	6.0%	FPG
					(age-adjusted prevalence)	
Mendes (2011) [26]	São Paulo, SP	2003	872	60+ years	17.9%	Self-report
Schmidt (2009) [28]	27 Brazilian	2006	54,369	aged ≥18 years	5.3%	Self-report
	state capitals					
Bosi (2009) [23]	São Carlos, SP	2007-2008	1,116	30-79 years	5% and 13.5%	OGTT and fasting capillary glycaemia
					(age-adjusted prevalence)	

^1^Data from publications of the Ministry of Health and other institutions were not included in the table.

^2^OGTT: oral glucose tolerance test (old diagnostic criteria for fasting glucose).

^3^FPG: fasting plasma glucose.

**Figure 1:**
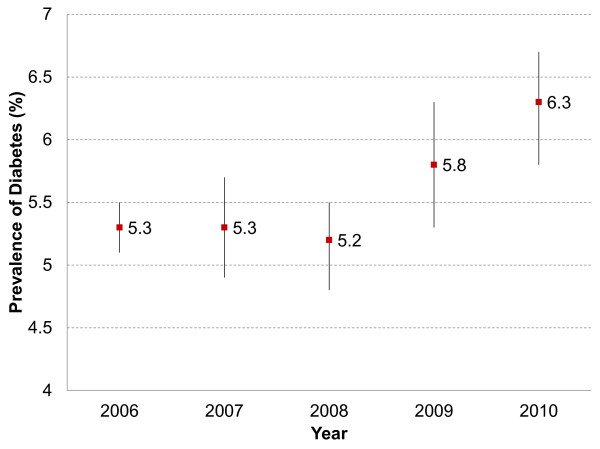
Prevalence of diabetes in Brazil between 2006 and 2010, according to the VIGITEL.

Self-reported prevalence of diabetes has been studied on an annual basis in all state capitals since 2006. As shown in Figure 1, within only four years, self-reported prevalence increased from 5.3% in 2006 to 6.3% in 2010. It is not clear whether this increase is due to increased prevalence, increased diagnosis or both.

**Figure 2:**
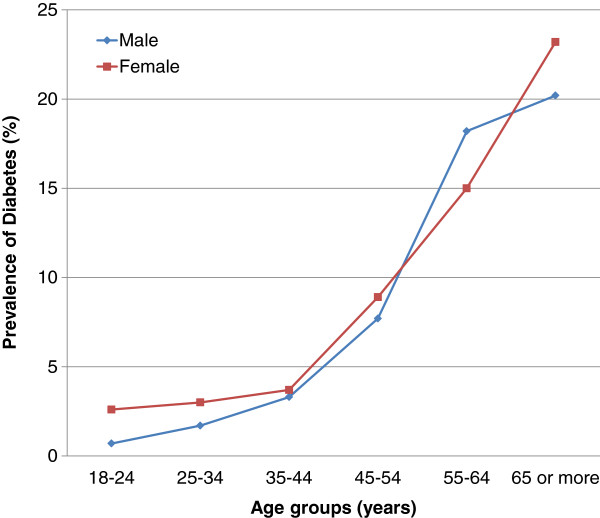
Prevalence of diabetes in Brazil by sex and age groups. VIGITEL, 2010.

As shown in Figure 2, women were more likely than men to report having diabetes, which may reflect their higher utilisation of medical care and therefore increased likelihood of being diagnosed [63], supporting the argument of increased detection. However, it seems likely that higher incidence of diabetes must also have played a role in increasing the reported prevalence of diabetes, particularly given the parallel increase in the prevalence of obesity epidemics in Brazil [72].

Franco et al. [73] analysed diabetes-relates deaths in São Paulo, including data from 1975 to 1992. Diabetes was mentioned on the certificate of 13,786 deaths (6.8%), and referred as the underlying cause of 2.6% of all deaths. Diabetes was also reported as an associated cause of deaths whose underlying cause was cardiovascular and respiratory diseases, as well as neoplasia.

Cesse et al. [18] analysed time trends in diabetes-related mortality and found that mortality increased in most state capitals between 1950 to 2000, while the largest proportional variations were observed in Teresina-PI (55.1%), Recife-PE (27%) and Natal (21.7%). This is consistent with the rapid demographic transition seen in Brazil during this period [2] as well as with the increased prevalence of diabetes. Mortality figures underestimate the burden of diabetes, since the underlying cause of death (including diabetes) is not accounted in the final cause of death estimates. Coeli et al. [19] examined 2,974 death certificates of older adults and found that 291 subjects had diabetes as one of the reported causes of death. However, only 150 subjects had diabetes as the underlying cause of death.

Diabetes was estimated to be responsible for 278,778 years of potential life lost for every 100,000 people [17]. Disability adjusted life years (DALYs) due to diabetes and its complications were estimated in the five regions of Brazil [20,22] – results showed that diabetes was responsible for 5.1% (6.0% among women and 4.4% among men) of the total DALYs in the country.

### Diabetes complications

It is estimated that about 7% of patients with diabetes had one or more of the following complications: diabetic foot ulcers, amputation, kidney disease, fundus changes [15]. Detailed information regarding studies on diabetes complication in Brazil is shown in Table 3.

**Table 3:** Studies on diabetes complications in Brazil

**First author (year)**	**City, State**	**N of patients with type 2 diabetes**	**Data collection**		**Complication**	**Prevalence**
			**Place**	**Source of data**		
Bruno (2000) [32]	Porto Alegre, Rio Grande do Sul	93	Dialysis centres	Standardized questionnaire, clinical interview, and review of medical records	Diabetic nephropathy	58.0%
					Diabetic retinopathy	85.0%
					Peripheral vascular disease	73.0%
Scheffel (2004) [37]	Rio Grande do Sul	927	Health care units	Clinical examination and laboratory tests	Coronary artery disease	36.0%
					Diabetic retinopathy	48.0%
					Ischemic heart disease	36.0%
					Peripheral vascular disease	33.0%
Tres (2007) [39]	Passo Fundo, Rio Grande do Sul	340	Outpatient Diabetes Clinic of Hospital São Vicente de Paulo	Questionnaire and neurological tests	Diabetic nephropathy	29.5%
					Diabetic neuropathy.	22.0%
					Diabetic retinopathy	28.8%
Vieira-Santos (2008) [40]	Recife, Pernambuco	1,374	Primary health care units	Medical records	Diabetic foot	9.0%

A study of 1,374 patients with diabetes seen in family health units in Recife, Pernambuco state found a 9% prevalence of diabetic foot [40]. Routine screening for diabetic foot is limited by the lack of trained podiatrists and appropriate supplies. With the exception of a few treatment centres, most health services, particularly primary health care, do not perform foot screening for patients at high risk of developing complications [36].

According to the Brazilian Ministry of Health, diabetic retinopathy (DR) is the leading cause of irreversible blindness in Brazil. Asymptomatic in its early stages, retinopathy evolves over time, affecting the majority of patients who have lived with diabetes for more than 20 years [21]. It is estimated that 20 to 40% of patients with type 2 diabetes are affected by DR, based on studies among specific groups and restricted areas [38,74-76]. The narrow focus of these studies and limited geographical coverage make it difficult to estimate the national prevalence.

Diabetic nephropathy (DN) is another common and devastating complication in patients with diabetes, with a slightly lower frequency than retinopathy [31]. Similar to other countries, chronic kidney disease has been an important public health problem in Brazil. It is estimated that at least one third of Brazilians with type 2 diabetes are affected by DN [34,35].

Data available from the High Complexity Procedures Authorisation Subsystem on Renal Replacement Therapy (APAC/TRS) [17] shows that between 2000 and 2006, 148,284 patients began dialysis treatments (predominantly haemodialysis) in Brazil. The incidence of terminal disease patients starting replacement therapy was estimated to be 119.8/1,000,000 inhabitants/year, varying from 143.6/1,000,000/year in the south of Brazil to 66.3/1,000,000/year in the north of the country [17,33]. Hypertension was reported as the leading cause of renal disease (22%), followed by diabetes mellitus (13.8%) and glomerulonephritis (7.2%) [17]. Undetermined causes were cited frequently (44.8%), indicating the need to improve the quality of the information recorded. Incidence of terminal disease patients starting dialysis increased in patients over 65 years, most likely related to population aging and greater use of renal replacement therapy among the elderly [33].

A population based study conducted in all 18 dialysis centres located in the metropolitan area of Porto Alegre between July 1995 and October 1996 followed 111 patients with type 2 diabetes for an average period of 3.6 years. The prevalence of DN was 58% and it was the leading cause of renal disease in 61% of all patients in the follow-up period (63%) [32].

Ischaemic heart disease and hypertension are the most frequent cardiovascular diseases in patients with diabetes. In women with diabetes, the protective effects observed for cardiovascular disease in general disappears [31]. In 2004, a cross-sectional study using a sample of 927 patients with type 2 diabetes treated at three medical centres in Rio Grande do Sul observed a prevalence of coronary artery disease, peripheral vascular disease and hypertension of 36%, 33%, and 73% respectively [37].

Regarding neuropathy, it is estimated that the most common form of the disease is distal symmetrical sensory polyneuropathy [15]. In 2007, a cross-sectional study with 340 patients with type 2 diabetes in Passo Fundo (southern Brazil) found a prevalence of 22% of diabetic peripheral neuropathy [39].

Despite the existence of multiple data sources, evidence on the prevalence and incidence of diabetes and its complications at national and regional level is very scarce and originates mainly from surveys. Prevalence data mainly originates from a number of studies that rely on self-reported data, and no study on the incidence of diabetes was found. It seems that there is a missed opportunity to leverage the data available through some of the national databases such as SUS and HiperDia, among others.

### Management of diabetes in Brazil: treatment, access, inequality

Evaluation of the health care delivered by SUS is still done infrequently, particularly with regard to chronic diseases. A study carried out by Assunção et al. [41] in 1998/1999 evaluated the structure, process, and outcomes of diabetes treatment in primary health care in Pelotas, in Southern Brazil. Approximately 85% of the physicians in the study reported prescribing a diet plan during their first consultation and 72% prescribed physical activity. In terms of laboratory monitoring of the patients, all physicians requested fasting blood glucose, while only 60% requested glycosylated haemoglobin.

In 2006, the Ministry of Health published primary health care guidelines [31] for the management of diabetes at primary care level. The guidelines provide recommendations on diabetes screening and prevention, diagnosis, initial evaluation and basic treatment. Screening is recommended for asymptomatic individuals at higher risk of diabetes according to the following indicators: age >45 years, BMI > 25 Kg/m^2^, waist circumference >102 cm for men and >88 cm for women, family history of diabetes, hypertension (>140/90 mmHg), HDL cholesterol <35 mg/dl and/or triglycerides >150 mg/dl. Recommendations on lifestyle changes, pharmacological treatment, prevention and management of acute and chronic outcomes of diabetes are also provided. Similar guidelines are available for hypertension and prevention of CVD at the primary care level.

Some studies using regional samples investigated availability, affordability and access to medicines used for the treatment of diabetes. Pinto et al. [45] analysed medicine prices and availability using WHO/HAI methodology. The study was performed in 2007 in 30 cities in Brazil and found that metformin 500 mg and glibenclamide 5 mg were available in 23% and 93% of public sector facilities respectively.

In contrast, another study [42] carried out in six cities in the south of Brazil found total availability of metformin 500 mg to be 100% in the public sector. In terms of affordability, the study found that both metformin and glibenclamide could cost up to two working days of salary for non-skilled workers to purchase a monthly course treatment. A cross-sectional study [43] evaluating 41 municipalities in South and Northeast Brazil reported that 78.6% of patients with diabetes had access to diabetes medicines. Another study using the same population [44] looked at access to diabetes medicine among the elderly and found that 95.8% had access to medicines, with the majority of medicines provided by SUS (76.7%).

A National Survey on Medicine Access and Utilization (PNAUM) started in 2013 and data collection is ongoing [77]. The aim of this survey is to evaluate the national pharmaceutical policy and whether the policy is achieving its main objective of ensuring high levels of access to medicine for the entire population. It is the first national study exclusively designed to evaluate the result of the current pharmaceutical policy.

The impact of diabetes on family expenses was investigated in a study using data from POF 2002-2003 [46]. This study showed that 1.7% of the population purchased at least one medicine for diabetes. The annual average spending for those who acquired one or more medicines for diabetes care amounted to USD $102.81.

Data from 2004 showed that glycaeted haemoglobin (HbA1c) control (<7.0%) was attained only by 40% of patients with diabetes [51].

### Diabetes outcomes indicators

Table 4 identifies indicators on diabetes outcomes available in Brazil. The main sources of data are information systems from the Ministry of Health. Minimal baseline information on each indicator is available.

**Table 4:** Indicators of diabetes outcomes available in Brazil

**Indicator**	**Source**
Percent of persons with diabetes mellitus with a HbA1c tested in last 12 months	Surveys
Percent of persons tested, who have HbA1c value >7.5%	Surveys
Percent of the persons with diabetes mellitus with microalbuminuria tested in last 12 months	Surveys
Percent of those tested with microalbuminuria	Surveys
Percent of the persons with diabetes mellitus with blood pressure measurements in last 12 months	SisHiperDia
Percent of the persons with diabetes mellitus who are smoking	SisHiperDia Vigitel
Percent of persons with diabetes mellitus with BMI ≥ 25 kg/m^2^, BMI ≥ 30 kg/m^2^	SisHiperDia Vigitel
Percent of persons with diabetes mellitus with fundus inspection in the last 12 months	SisHiperDia
Percent of those tests, with proliferate retinopathy in the last 12 months	SisHiperDia
Annual incidence of amputations above the ankle in patients diabetes mellitus/100,000 general population	Surveys
Annual incidence of myocardial infarction in patients with diabetes mellitus/100,000 general population	SisHiperDia

Data from 2004 showed that glycaeted haemoglobin (HbA1c) control (<7.0%) was attained only by 40% of patients with diabetes [51]. Further, it is estimated that about 7% of individuals with diabetes had one or more of the following complications: diabetic foot ulcers, amputation, kidney disease, fundus changes [15].

A multicentre study conducted in five countries, including Brazil, identified that no country has reached the standard for HbA1c or blood pressure set by the American Diabetes Association Diabetes Physician Recognition Programme [52]. In 2007, a cross-sectional multicentre study conducted in nine Latin American countries (Argentina, Brazil, Chile, Costa Rica, Ecuador, Guatemala, Mexico, Peru, and Venezuela), including a sample of 878 Brazilians aged 18 to 75 years with type 2 diabetes, showed that about 40% of participants had controlled glycosylated haemoglobin (HbA1c <7.0%) [51].

Very few studies have been conducted to evaluate the quality of treatment and to measure differences between SUS and privately insured patients. A retrospective cohort study was carried out in Southern Brazil involving 80 patients treated in a SUS outpatient clinic and 277 patients treated at a private clinic. Patients receiving treatment from SUS generally showed worse metabolic control, although only the values of HbA1c and total cholesterol were statistically different between the two groups [50]. However, due to the small sample size and the regional coverage of this study, these findings are not representative of the whole Brazilian population.

### Costs related to diabetes and its complications

In 2008, the World Bank estimated that countries such as Brazil, China, India and Russia lose more than 20 million productive life-years due to NCDs annually [78].

A study across several Latin and Central American and Caribbean countries [79] estimated that in 2000, the total annual costs (direct and indirect) of diabetes in Brazil were USD $22.6 billion. Direct costs included medications, hospitalisations, consultations, and treatment for complications and totalled to US $3.952 billion. This represented a direct cost per capita of US $872. Indirect costs included loss of income by permanent and temporary incapacity as well as premature death, and amounted to USD $18.6 billion. Across all twenty-five Latin American and Caribbean countries included in the analysis, Brazil had the highest estimated indirect and direct costs for diabetes among the countries studied.

Bahia et al. [54] estimated direct and indirect costs of type 2 diabetes using data collected during 1,000 interviews carried out in 2007 in eight Brazilian cities. The total annual cost per patient was USD $2,108, of which 63.3% were direct costs (USD $1,335) and 36.7% indirect costs (USD $773).

McLellan et al. [55] estimated the cost of clinical treatment and hospital expenses to be around USD $710 per patient/year in 2001. This estimate was based on 93 people with diabetes in the city of Piracicaba - São Paulo - hospitalised between March and June 2001, and therefore unlikely to be nationally representative.

Rosa et al. [57] calculated expenses for hospitalisation due to diabetes using national data for the period of 1999–2001. It was estimated that the average cost per hospitalisation resulting in patient death is USD $275.27; in comparison to USD $143.45 when hospitalisation did not result in death [57]. Hospitalisation rates for patients with diabetes have been stable in the past few years, ranging from 65 to 75 per 100,000 inhabitants per year.

Abegunde et al. [53] predicted that losses due to reduced productivity at work and the decreased family income as a result of diabetes, heart disease and stroke would lead to an economic loss amounting to USD $4.18 billion from 2006 to 2015 in low and middle-income countries.

A study using DATASUS data estimated the direct cost of hospitalisation due to diabetes to be USD $362,945,412 in 2000 [58]. Another study [56] simulated a hypothetical cohort including 6.48 million participants with type 2 diabetes, based on estimates from the Brazilian Ministry of Health, hospital budgets and expense records in 2008. The estimated annual total cost of hospitalisation was USD $264 million (converted using 2008 rate of exchange US$1 = R$1.64), while the costs related to amputation totalled USD $128 million [56].

### Health policy related to diabetes

In 1987, a multicentre study on the prevalence of diabetes and impaired glucose tolerance was conducted in nine Brazilian capitals among adults aged 30–69. This study indicated that half of the individuals with diabetes were not aware of their health condition [21].

In an attempt to address the high level of unawareness about diabetes, the first national diabetes screening campaign was launched in 2001 and implemented by public health services in Brazil. The target population was SUS users aged 40 years or older. The estimated national coverage of the campaign among the SUS target population was 73% [80]. Twenty million people were screened using capillary glycaemia tests and approximately 3.3 million (16.5%) suspected cases of diabetes were identified [81].

The Primary Health Care Department within the Health Care Secretariat develops measures to control and assess services from the primary health care and provides technical support to states, cities, and the Federal District. The Department organises basic health services including the Family Health Programme (PSF), oral health, hypertension and diabetes (HiperDia), food and nutrition, management and strategies, evaluation and follow-up activities [81].

According to the guidelines from HiperDia, risk prevention and care of patients with diabetes should take place at primary health care level [17]. The Family Health Strategy [5] was introduced in 1994, aiming to reorganise primary health care through the implementation of multi-disciplinary professional teams. These teams are responsible for the follow-up of a defined number of families located in a limited geographical area. The teams work on health promotion actions, prevention, recuperation, rehabilitation, and the maintenance of community health. The strategy aims to rationalise the use of all levels of assistance (primary, secondary and tertiary) and it has produced positive results for the main health indicators in the populations benefitting from the family health teams.

SUS provides essential medicines for diabetes control without additional costs for the system’s users. The free distribution of medicines in Brazil began in 1971, focusing on the poor population [82]. The Brazilian programme Popular Pharmacy was created in 2004 as a partnership between the federal government and states/municipalities aiming at increasing access to low-cost essential medications for the Brazilian population [82]. In 2006, this strategy was expanded to include private pharmacies and drug stores, named “*Aqui Tem Farmácia Popular*” (Popular Pharmacy is Available Here) [82]. As part of this programme, the Ministry of Health began subsidising 90% of the reference price of 24 medicines for the treatment of hypertension, diabetes, asthma, rhinitis, Parkinson disease, osteoporosis and glaucoma. This programme covers more than 2,500 municipalities and is available to 1.3 million Brazilians in need of medication (patients for whom drugs were prescribed), including 300,000 patients with diabetes [17].

In September 2006, a law was enacted to ensure the free distribution of diabetic medicines and the necessary equipment to monitor capillary glycaemia for all SUS insurees. In 2007, it was established that free medicines would be available to patients with diabetes, although the free distribution was restricted to patients whose treatment was provided by the SUS in primary health care units. In March 2011, the Brazilian government launched a programme called *“Saúde Não Tem Preço”* (Health has no price), to expand access to medicines for diabetes and hypertension. In this programme, the pharmacies and drugstores linked to the popular pharmacy network started to offer free medicines for the treatment of hypertension and diabetes (glibenclamide, metformin and insulin) in more than 17,500 registered private pharmacies [17]. A month after its launch, more than 3.7 million treatments were distributed, representing an increase of 70% in the distribution of medicines for hypertension and diabetes [17].

Brazil has participated in health promotion campaigns related to diabetes such as World Diabetes Day. The main strategy adopted by the Government to prevent chronic diseases is to control risk factors. Preventive efforts include anti-tobacco programmes, food and health nutrition policies (industry self-regulation code on advertising of food and beverages directed at children, regulation requiring the inclusion of warnings in all forms of advertising for products containing high levels of fat, sugars or salt), school health promotion, and actions to ensure essential medicines are provided in the public sector for hypertension and diabetes [17,83].

The Health Gym Programme was created in order to promote physical activity and provide free-of-charge spaces and support for living a healthy lifestyle [17]. According to the strategic action plan for coping with chronic diseases in Brazil from 2011 to 2022, the programme’s goal is to reach 4,000 municipalities by 2015 [84].

### Actions for the future

Recently the Brazilian Ministry of Health launched the National Strategy for the Prevention and Control of NCDs for the period 2011–2022 [17]. The plan aims to prepare Brazil to confront and prevent the major chronic NCDs in the next ten years.

The Brazilian National Policy on Health Promotion [85] has prioritised drafting regulatory measures aimed at promoting healthy eating to reduce the prevalence of NCDs, with special emphasis on the regulation of food marketing and advertising, encouraging physical activity through gym classes at community levels, and implementing health promotion strategies in schools.

The expansion of pharmaceutical care and the free distribution of more than 15 medications for hypertension and diabetes play an important role in the Brazilian Government’s effort to tackle diabetes. In September 2011, Brazilian President Dilma Rousseff attended a general assembly summit at the UN headquarters in New York, contributing to global efforts in confronting the problem of NCDs [6]. The President reported that one of the first measures of her government was to increase access to medicines for poor patients with hypertension and diabetes. According to the President, 5.4 million Brazilians have taken advantage of the programme.

## Conclusions

According to the latest IDF estimates, the prevalence of diabetes in Brazil was 10.3% in 2012. However, this national level estimate hides important intra-country variation.

In the last few years, the Brazilian Ministry of Health has invested considerably in surveillance systems on NCDs. As a result, our review identified a number of data sources relevant to the study of diabetes covering morbidity (SIH-SUS HiperDia), mortality (SIM), risk factors (VIGITEL, ELSA), access and utilisation of health care services (PNAD, POF). However, it seems that the country is still not capitalising on available national data to produce the necessary evidence to identify gaps and formulate appropriate policy responses.

Data on diabetes costs are patchy and out-of-date. A multicountry study estimated that the total annual costs (direct and indirect) of diabetes in the country were USD $22.6 billion in 2000, representing a direct cost per capita of US $872. A more recent study estimated the direct and indirect costs of diabetes to be USD $ 2,108 per capita in 2007. There is some evidence on hospitalisation costs but no evidence on the cost of various types of complications.

A number of policies and programmes have been introduced by the Brazilian government in an attempt to improve access to diabetes care and reduce the prevalence of the disease. These include a national diabetes screening campaign in 2001, the Brazilian Popular Pharmacy programme introduced in 2004 and preventive efforts addressing risk factors (regulation of the food industry, promotion of physical activity through the health gym programme and anti-tobacco programmes).

Considering the magnitude of diabetes in Brazil, the Ministry of Health has adopted several strategies to reduce the costs of the disease in the Brazilian population, highlighting the interventions to be taken at the primary health care level. Specific programmes were implemented aimed at managing diabetes. However, some of the gaps include weak evaluation of the SUS in providing good quality care for patients with diabetes and lack of data on inequalities in access to medicines and health care services including annual testing for complications.

In conclusion, Brazil has the capacity to address and respond to NCDs due to the availability of federal, state and local integrated health programmes currently in operation. There is funding available for NCDs treatment, control and prevention, as well as health promotion, surveillance, monitoring and evaluation activities. However, these resources need to be used in the right way to be effective.

## Abbreviations

AIH: (*Autorização de Internação Hospitalar) -* Hospital Admission Authorization Form; APAC: (*Autorização de Procedimentos de Alta Complexidade)* - Authorization for Procedures of High Complexity SUS; CNG: (*Glomerulonefrite crônica)* - Chronic glomerulonephritis; CKD: (*Doença renal crônica*) - Chronic kidney disease; DAB: (*Departamento de Atenção Básica*) - Primary Health Care Department; DALYs: (*Anos de vida ajustados para incapacidade)* - Disability adjusted life years; DATASUS: (*Banco de Dados do SUS*) – SUS Dataset; DHS: (*Pesquisa de Demografia e Saúde)* - Demographic and Health Survey; DM: (*Diabetes Mellitus)* - Diabetes Mellitus; DR: (*Retinopatia Diabética*) - Diabetic Retinopathy; ELSA: (*Estudo Longitudinal de Saúde do Adulto)* - Adult Health Longitudinal Study; ESF: (*Estratégia Saúde da Família)* - Family Health Strategy; GDP: (*Produto Interno Bruto)* - Gross Domestic Product; HAS: (*Hipertensão Arterial Sistêmica)* - Hypertension; HbA1c: Glycaeted Haemoglobin; HiperDia: (*Sistema de cadastramento e acompanhamento de hipertensão e diabetes)* – Hypertension and Diabetes Registration and Follow-up System; IBGE: (*Instituto Brasileiro de Geografia e Estatística)* - National Institute of Geography and Statistics; NCDs: (*Doenças crônicas não transmissíveis)* - Non-communicable diseases; PNAD: (*Pesquisa Nacional de Amostra de Domicílios)* - National Household Sample Survey; PNAUM: (*Pesquisa Nacional sobre Acesso, Utilização e Promoção do Uso Racional de Medicamentos no Brasil)* - National Research of Medicine Access and Utilization; PNDS: (*Pesquisa Nacional de Demografia e Saúde)* - National Demography and Health Survey; PNPS: (*Política Nacional de Promoção da Saúde*) - Brazilian National Policy on Health Promotion; PNS: (*Pesquisa Nacional de Saúde)* - National Health Research; POF: (*Pesquisa de Orçamentos Familiares)* - Family Budget Survey; PROESF: (*Projeto de Expansão e Consolidação Saúde da Família)* - Family Health Expansion and Consolidation Project; PSF: (*Programa Saúde da Família)* - Family Health Programme; SAMHPS: (*Sistema de Assistência Médico-Hospitalar da Previdência Social)* - Social Security Medical Assistance System; SIA/SUS: (*Sistema de Informações Ambulatoriais*) – Ambulatory Information System; SIH/SUS: (*Sistema de Informações Hospitalares do SUS*) - Hospital Information System; SAS: (*Secretaria de Atenção à Saúde*) – Health Care Secretariat; SIAB: (*Sistema de Informação da Atenção Básica*) – Primary Health Care Information System; SIM: (*Sistema de Informação de Mortalidade*) - Mortality Information System; SIS/HiperDia: (*Sistema de Informação do HiperDia*) - HiperDia system; SUS: (*Sistema Único de Saúde*) - Unified Health System; WHO: (*Organização Mundial da Saúde*) – World Health Organization; USAID: (*Agência dos Estados Unidos para o Desenvolvimento Internacional*) - http://www.usaid.gov/; VIGITEL: (*Vigilância de Fatores de Risco e Proteção para Doenças Crônicas por Inquérito Telefônico*) - Surveillance System of Risk and Protective Factors for Chronic Non-Communicable Diseases through Telephone Interviews.

## Competing interests

The authors declare that they have no competing interests. The funding to conduct this study was provided by Novo Nordisk Switzerland. The sponsor had no involvement in the study design, data collection and analysis, and writing. AF received travel reimbursement and speaker fees from Novo Nordisk for delivering two presentations on diabetes in EU5 (France, Germany, Italy, Spain and UK) at national diabetes conferences in Portugal and Spain.

## Author’s contributions

ADB was the main investigator involved in the acquisition of data and drafting the manuscript. PK coordinated the conception, design and interpretation of data. GVAF participated in the acquisition of data and in drafting the manuscript. AC and CAOT were involved in the acquisition of specific data and drafting part of the manuscript. PCH, MIS and AF revised the manuscript critically for important intellectual content. All authors read and approved the final version of the manuscript to be published.
